# NEK2 contributes to radioresistance in esophageal squamous cell carcinoma by inducing protective autophagy via regulating TRIM21

**DOI:** 10.1186/s12935-024-03367-5

**Published:** 2024-05-23

**Authors:** Dong Guo, Shuo Zhou, Ruixue Liu, Weinan Yao, Shuguang Li, Xueyuan Zhang, Wenbin Shen, Shuchai Zhu

**Affiliations:** Department of Radiation Oncology, Fourth Hospital of Hebei Medical University, Shijiazhuang, 050000 China

**Keywords:** NEK2, Radioresistance, Autophagy, TRIM21, Esophageal squamous cell carcinoma

## Abstract

**Background:**

Radiotherapy (RT) has been identified as a vital treatment for esophageal squamous cell carcinoma (ESCC), while the development of radioresistance remains a major obstacle in ESCC management. The aim of this study was to investigate the effect of NIMA-related kinase 2 (NEK2) on radioresistance in ESCC cells and to reveal potential molecular mechanisms.

**Methods:**

Human esophageal epithelial cells (HEEC) and human ESCC cell lines were obtained from the Research Center of the Fourth Hospital of Hebei Medical University (Shijiazhuang, China). Cell Counting Kit-8 (CCK-8) and flow cytometry assays were applied to assess the proliferation ability, cell cycle, apoptosis rates, and ROS production of ESCC cells. The colony-forming assay was used to estimate the effect of NEK2 on radiosensitivity. Autophagy was investigated by western blotting analysis, GFP-mRFP-LC3 fluorescence assay, and transmission electron microscopy (TEM).

**Results:**

In the present study, our results showed that NEK2 was associated with radioresistance, cell cycle arrest, apoptosis, ROS production, and survival of ESCC. NEK2 knockdown could significantly inhibit growth while enhancing radiosensitivity and ROS production in ESCC cells. Interestingly, NEK2 knockdown inhibited ESCC cell autophagy and reduced autophagic flux, ultimately reversing NEK2-induced radioresistance. Mechanistically, NEK2 bound to and regulated the stability of tripartite motif-containing protein 21 (TRIM21). The accumulation of NEK2-induced light chain 3 beta 2 (LC3B II) can be reversed by the knockdown of TRIM21.

**Conclusion:**

These results demonstrated that NEK2 activated autophagy through TRIM21, which may provide a promising therapeutic strategy for elucidating NEK2-mediated radioresistance in ESCC.

## Introduction

Esophageal cancer is an aggressive gastrointestinal cancer and ranks as the sixth leading cause of cancer-related mortality globally [1, 2]. Esophageal squamous cell carcinoma (ESCC) is the predominant histological subtype in Asian countries, where ESCC accounts for over 80% of all esophageal cancers [3]. Even with advanced instrumentation and multidisciplinary discussions, the therapeutic benefit is unsatisfactory for most ESCC patients diagnosed at middle or late stages [4]. Radiotherapy has been applied as the effective treatment for these ESCC patients. Unfortunately, more than 50% of ESCC patients do not show adequately treatment response and encounter recurrence as a result of tumor radioresistance [5]. There are many factors that contribute to the development of resistance to the treatment of tumors [6, 7]. Therefore, elucidating the underlying mechanisms behind radioresistance and overcoming radioresistance are urgently required to develop effective strategies for ESCC patients.

NIMA-related kinase 2 (NEK2) is a serine/threonine kinase known to localize to the centrosome and functions as a member of the NIMA-related kinase family [8]. Previous data indicated that NEK2 played a vital role in various biological functions including tumor proliferation, epithelial-mesenchymal transition (EMT), drug resistance, oncogenesis, and immune regulation [9, 10]. Studies have shown that NEK2 was commonly overexpressed in malignant tumors [11–13]. In our prior research, we demonstrated experimentally that the overexpression of NEK2 potentially led to tumor development and predicted a poor prognosis in ESCC patients [14]. Recently, a study revealed an association between NEK2 and radioresistance in cervical cancer, showing its influence on cellular sensitivity to ionizing radiation (IR) by regulating DNA damage and apoptosis [15]. Further mechanistic studies indicated that NEK2 induced radioresistance by destabilizing the histone methyltransferase EZH2 in glioblastoma cells [16]. Nonetheless, the molecular mechanism of NEK2 conferring radioresistance in ESCC has not been elucidated.

Autophagy is a conserved metabolic degradation process by which cells can maintain genomic integrity and homeostasis through intracellular clearance and recycling [17]. During stress conditions, damaged or redundant cellular components are sequestered by autophagosomes and delivered to the lysosome, where they are degraded to adapt cellular conditions for survival. Interestingly, previous data has reported that autophagy can act as both a tumor suppressor and a tumor activator, thereby presenting dual roles [18, 19]. More recently, studies indicated that the autophagy can regulate cellular sensitivity to IR and activation of autophagy brought back radioresistance [20, 21]. Thus, we hypothesized that autophagy was associated with radioresistance in ESCC and inhibition of autophagy may overcome NEK2-mediated radioresistance.

In the present study, we found that NEK2 could promote autophagy level in ESCC cells. The tripartite motif-containing protein 21 (TRIM21) was identified as a key modulator of NEK2-induced autophagy. Inhibitor of autophagy or TRIM21 can overcome NEK2-mediated radioresistance. These findings have identified potential therapeutic targets for enhancing sensitivity to IR in ESCC patients.

## Materials and methods

### Data collection preprocessing

The raw intensities of mRNA information derived from the GSE161533, GSE20347 and GSE53624 datasets were downloaded from the Gene Expression Omnibus (GEO) database (https://www.ncbi.nlm.nih.gov/geo/). Gene expression data was used to analyze the differential expression of target genes in between ESCC and normal tissues. Then we used GraphPad software to plot the overall survival (OS) survival curve.

### Antibodies and reagents

Antibodies against CDK1 (ab133327), Cyclin B1 (ab32053) and CDC25C (ab32444), Caspase-3 (ab32351), Bcl2 (ab182858), LC3B (ab192890), SQSTM1/P62 (ab109012), and γH2AX (ab81299) were obtained from Abcam (Cambridge, MA, USA). Antibodies against NEK2 (66632-1-lg), TRIM21(12108-1-AP), α-tubulin (11224-1-AP), GAPDH (60004-1-Ig), Bax (60267-1-Ig), Alexa Fluor 594-conjugated donkey anti-rabbit second antibody (SA00013-4), and Alexa Fluor 488-conjugated donkey anti-mouse second antibody (SA00013-1) were obtained from Proteintech (Wuhan, China). An antibody against NEK2 (sc55601) was got from Santa Cruz Biotechnology, Inc (St Louis, MO, USA). The autophagy activator Rapamycin (RAPA, HY-10,219), Cycloheximide (CHX, HY-12,320), and MG132(HY-13,259) were purchased from MedChemExpress (MCE, USA).

### Cell culture and irradiation

Human normal esophageal epithelial cells (HEEC) and Human ESCC cell lines, including ECA109, TE1, KYSE30, KYSE150, KYSE410, and KYSE450, were obtained from the Research Center of the Fourth Hospital of Hebei Medical University (Shijiazhuang, China). Cells were cultured in RPMI 1640 medium supplemented with 10% fetal bovine serum (FBS) and 1% penicillin and streptomycin at 37 °C incubator containing 5% CO^2^. The medium of cell lines was refreshed every 2 to 3 days. ESCC cells were exposed to X-ray irradiation by adopting the single energy 6-MV Siemens linear accelerator (Siemens, Buffalo Grove, IL, USA) at a 3 Gy/min dose-rate.

### Plasmids and lentiviral construction

In order to obtain NEK2 overexpressing cells, full-length human NEK2 cDNA was amplified and cloned. NEK2 shRNA and adenovirus expressing mCherry-GFP-LC3 fusion protein lentivirus were purchased from Shanghai Genechem Company Co. Ltd (Shanghai, China). The siRNA targeting TRIM21 was synthesized by Ribo BioTechnology (Guangzhou, China). Adherent cells were infected with shRNA adenovirus according to the manufacturer’s instructions. The stable cell lines were generated after puromycin (2 µg/L) selection media.

### Quantitative real-time PCR (qRT-PCR)

RNA was extracted from ESCC cells using Trizol reagent (Thermo Fisher Scientific, Inc.). Subsequently, the total RNA was reverse transcribed to cDNA using 20 µL of the reverse transcription kit reaction reagents (Thermo Fisher Scientific, Inc. cat. K1622) following the manufacturer’s instructions. MonAmp™ SYBR® Green qPCR Mix (Monad Biotech Co., Ltd.) was utilized for qRT-PCR to analyze the expression level of gene. GAPDH was used as an internal control to normalize the targeting gene expression levels. Relative NEK2 expression level was calculated by 2^−ΔΔCt^ method.

### Western blotting assay

The specific procedure was performed as previously described [20]. In brief, the extracted total protein was separated using an SDS-PAGE gel and transferred to polyvinylidene fluoride (PVDF) membranes. After blocked with 5% skim milk, the PVDF membranes were incubated with the primary antibodies overnight at 4 °C, and followed by horseradish peroxidase-conjugated secondary antibodies at room temperature for 1 h. The immunoreactive bands of each protein were visualized by an Odyssey Infrared Imaging System (LI-COR Biosciences, Lincoln, NE, USA), and band quantification was analyzed using ImageJ software.

### Subcellular fractionation assay

Nuclear protein and cytoplasmic protein extraction Kit (Proteintech, Wuhan, China) was applied to conduct the subcellular fractionation assay according to the manufacturer’s instructions. The western blotting assay was used to analyze the protein expression in nuclear and cytoplasmic. These experiments were performed three times.

### Cell viability assay

The cells with NEK2 knockdown were seeded into 96-well plates with 4 × 10^3^ cells per well. Briefly, the cell viability was examined at different time points (0 h, 24 h, 48 h, 72 h and 96 h) using 10 µL CCK-8 solution (Med Chem Express (MCE) Princeton, NJ, USA) in each well. After 2 h incubation, the absorbance at 450 nm was measured using a microplate reader and the cell growth curve was plotted with 5 independent replicates.

### Plate colony formation assay

The ESCC cells were seeded in 6-well plates and were irradiated with 0, 2, 4, 6, or 8 Gy X-ray beam dose. After 14 days of.

culture, cells were fixed with 4% paraformaldehyde for 20 min and stained with 0.1% crystal violet for 20 min, then gently washed with water for colony counting. The number of cells between knockdown and NC cells at 0 Gy IR treatment was normalized. The dose-response curves were calculated using the single-hit multitarget model. The Formula [SF = 1 - (1 - e-D/D0) N] was applied for this model.

### Apoptosis assay

The Apoptosis assay was performed using Annexin V- fluorescein isothiocyanate (FITC)/7-AAD Apoptosis Detection Kit (BD Biosciences, San Jose, CA, USA) according to the manufacturer’s protocol. Briefly, cells of each sample were trypsinized with 0.25% trypsin and resuspended with PBS three times. Subsequently, 5 µl of Annexin V- FITC and 5 µl of PI solution was mixed with 300ul binding buffer. The Annexin V- FITC/7-AAD was analyzed by flow cytometry (Beckman, USA), and apoptosis rates were calculated using FlowJo software.

### Cell cycle assay

ESCC cells were incubated in 6-well plates at the concentration of 3 × 10^5^ cells/well and irradiated with 6 Gy X-ray beam dose for 48 h. Subsequently, cells were trypsinized and washed twice with PBS. The cells were stained with a solution containing propidium iodide (PI) and DNase-free RNase A (Sigma-Aldrich). The data representing cells in each phase of the cell cycle was analyzed using FlowJo V10 cytometry software.

### Intracellular reactive oxygen species (ROS) detection

Intracellular ROS level was assessed in live cells using DCFH-DA (Molecular Probes, Beyotime, Shanghai, China). The cells grown in 6-well plates were treated with or without irradiation, then pre-incubated with 10 µM DCF-DA reagent for 30 min at 37 °C. The intensity of mean fluorescence was immediately determined for cells via flow cytometry at specific wavelengths (excitation wavelength: 488 nm; emission wavelength: 525 nm).

### Neutral comet assay

To assess the level of DNA double-strand breaks (DSBs) in cells, comet assays were performed using a commercial kit (IPHASE, China). According to the manufacturer’s instructions, cell suspension was mixed 1:10 with low melting point agarose and immobilized on the initial preparation of comet slide with normal melting point agarose. After overnight lysis, cells were subjected to neutral electrophoresis at 23 V for 30 min. The cells were stained with SuperRed (Seven) and imaged using a fluorescence microscope (Nikon A1, Japan). The mean olive tail moments were calculated by the CometScore 2.0 software.

### Immunofluorescence assay

2 × 10^4^ cells were plated at 15-mm confocal dishes and fixed with 4% formaldehyde. Then, the 0.3% Triton X-100 was used to permeabilize the cells for 10 min and blocked in 10% goat serum (Beyotime) for 30 min. The blocked cells were incubated with diluted primary antibodies of NEK2, γH2AX, LC3B and TRIM21 overnight at 4°C. Next, the confocal dishes were washed with PBS three times, followed by incubation with secondary antibodies conjugated with Alexa Fluor 594 goat anti-rabbit antibody and Alexa Fluor 488 goat anti-mouse antibody for 1 h at room temperature. Thereafter, the nucleuses were labeled with DAPI solution (Invitrogen). Cell samples were visualized using a fluorescence microscope (Zeiss LSM900, Germany).

### Co-immunoprecipitation (Co-IP)

Cell protein was extracted with RIPA lysis buffer (Thermo Fisher Scientific, USA). Cell lysates were incubated with indicated antibodies overnight at 4 °C. Then, the immune complexes were precipitated with protein A/G agarose beads (MCE, HY-K0202, USA) for 4 h at 4 °C. After washing beads with TP-40, the pulled down proteins were subjected to western blotting.

### Transmission electron microscopy (TEM)

TEM was carried out to analyze the ultrastructural autophagosomes. Cells were fixed in a solution containing 2.5% glutaraldehyde and 2% paraformaldehyde in 0.1 M cacodylate buffer for 2 h. The fixed cells were then post-fixed with 1% oso4 for 1.5 h. The cell samples were dehydrated with graded alcohol and embedded in Epon-Araldite resin. Ultrathin sections were obtained and placed on a support membrane for further observation using TEM HT7800 (Hitachi, Tokyo, Japan).

### Autophagy flux assay

To monitor autophagy flux, the cells were infected with GFP-mRFP-LC3 double-labeled lentiviral system (GeneChem, China) according to the manufacturer’s instruction. 1 × 10^4^ cells were planted in 15-mm confocal dishes and exposed to irradiation with a single 6 Gy dose. After 48 h, the autophagy flux was visualized with ZEISS LSM900 Confocal Laser Scanning Microscope (ZEISS, Germany).

### Xenograft tumor models

All animal experiments were conducted with the approval of the Animal Care and Use Committee of the Fourth Hospital of Hebei Medical University. Mice were obtained from Huafukang Animal Technology Co., Ltd. (Beijing, China). Male BALB/c nude mice were injected with 1 × 10^7^ TE1-NC cells or 1 × 10^7^ TE1-shNEK2 cells resuspended in 200 µL PBS. The BALB/c nude mice were maintained in a stable environment (23 °C) for one week before experiments. IR treatment was started seven days after transplantation. The tumor’s length (a) and width (b) were measured every three days and the tumor volume was calculated using the formula V = ab^2^/2. After 15 days, mouses were sacrificed and tumors were stripped and weighed.

### Statistical analysis

Data analysis was conducted using GraphPad Prism 8.0 (GraphPad Software, Inc., CA, USA) or R software version 3.5.2. The results were presented as the mean ± standard deviation (SD) of three experiments, and the differences between two groups were analyzed using a two-tailed Student’s t-test, while variations among several group means were determined using one-way ANOVA. Statistical significance was considered as a p value less than 0.05.

## Results

### NEK2 was overexpressed in ESCC and was associated with a poor outcome for ESCC patients

**Fig. 1:**
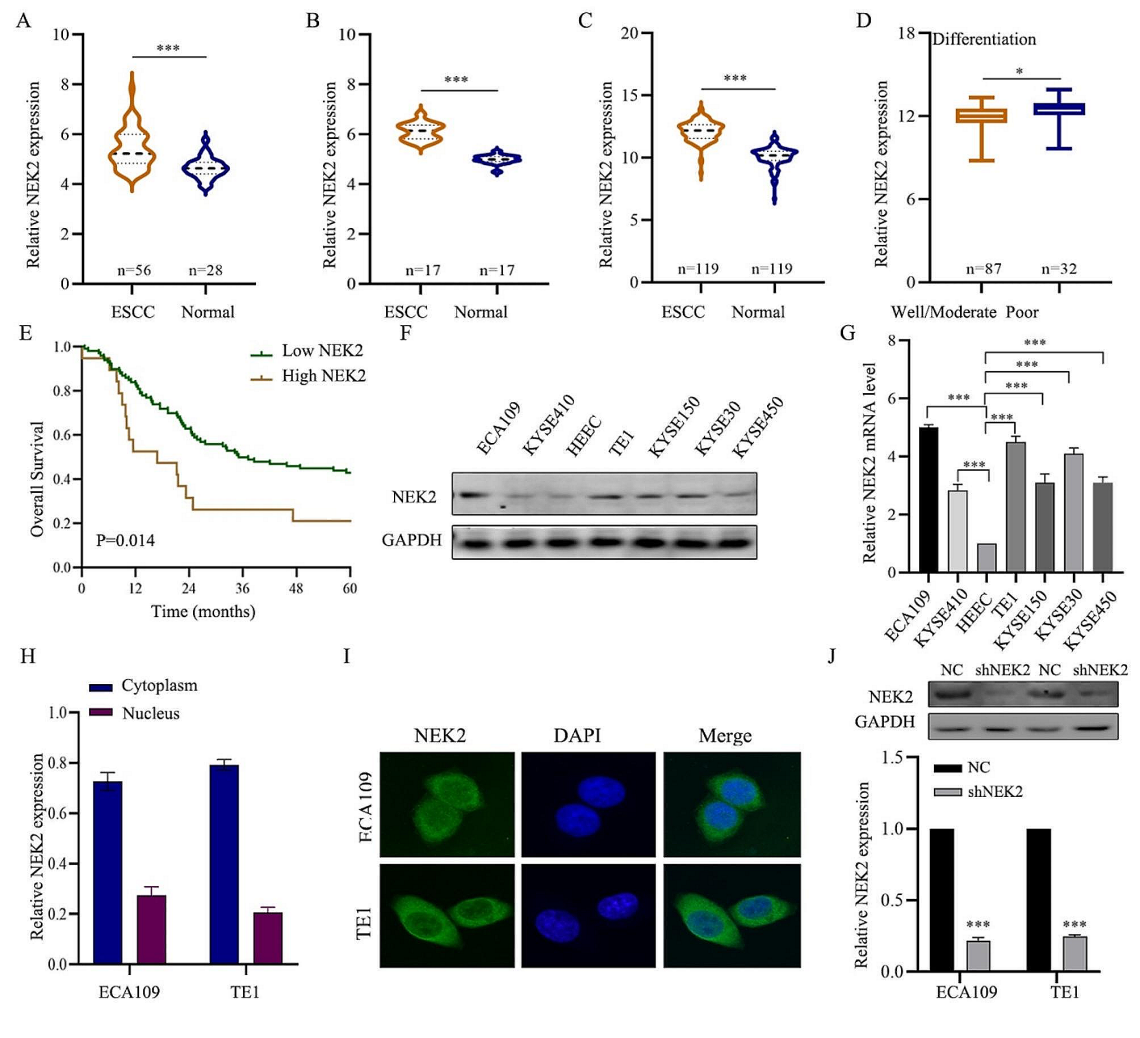
NEK2 expression up-regulated, and its expression is associated with unfavorable prognosis of ESCC patients. **A-C** Different expression levels of NEK2 in GEO cohorts of GSE161533, GSE20347 and GSE53624. **D** ESCC patients with poor differentiation showed the highest NEK2 expression. **E** Kaplan-Meier analysis of NEK2-related OS curves in ESCC patients. **F, G** The NEK2 expression level of HEEC and six ESCC cells were assessed by western blotting assay and qRT-PCR assay. **H, I** Immunofluorescence assay testing the location of NEK2 in ESCC cells. **J** The knockdown efficiency by transfection of shNEK2 was validated by western blotting assay and qRT-PCR. *p < 0.05, ** *p* < 0.01, *** *p* < 0.001

We firstly validated the NEK2 expression in ESCC based on GEO datasets (GSE161533, 56 ESCC vs. 28 normal tissues; GSE20347, 17 ESCC vs. 17 normal tissues; GSE53624, 119 ESCC vs. 19 normal tissues). Using three GEO datasets, the results revealed that NEK2 was significantly up-regulated in ESCC tissues compared with normal esophageal tissues (Fig. 1A-C). In the subgroup analysis of differentiation within the GSE53624 cohort, NEK2 was significantly higher in the poorly differentiated subtype compared to the well differentiated and moderately differentiated subtypes (*p* < 0.05; Fig. 1D). Furthermore, survival analysis indicated that NEK2 was a unique molecule associated with a poor outcome in ESCC patients (*p* = 0.014; Fig. 1E).

We investigated the NEK2 protein expression in HEEC and ESCC cell lines, including ECA109, TE1, KYSE30, KYSE150, KYSE410, and KYSE450. The western blotting results indicated that the NEK2 expression in ECA109 and TE1 cells were higher than that in other ESCC cell lines (Fig. 1F). Consistently, the mRNA results confirmed the previous protein expression results (Fig. 1G). To obtain more information about the localization of NEK2, the protein expression and immunofluorescence results showed that NEK2 was predominantly located in the cytoplasm in the ECA09 and TE1 ESCC cell lines (Fig. 1H-I). Additionally, the NEK2 knockdown efficiency was validated by qRT-PCR and western blotting assay (Fig. 1J).

### **NEK2 knockdown promoted IR-induced cell cycle arrest and spindle microtubules confusion**

**Fig. 2:**
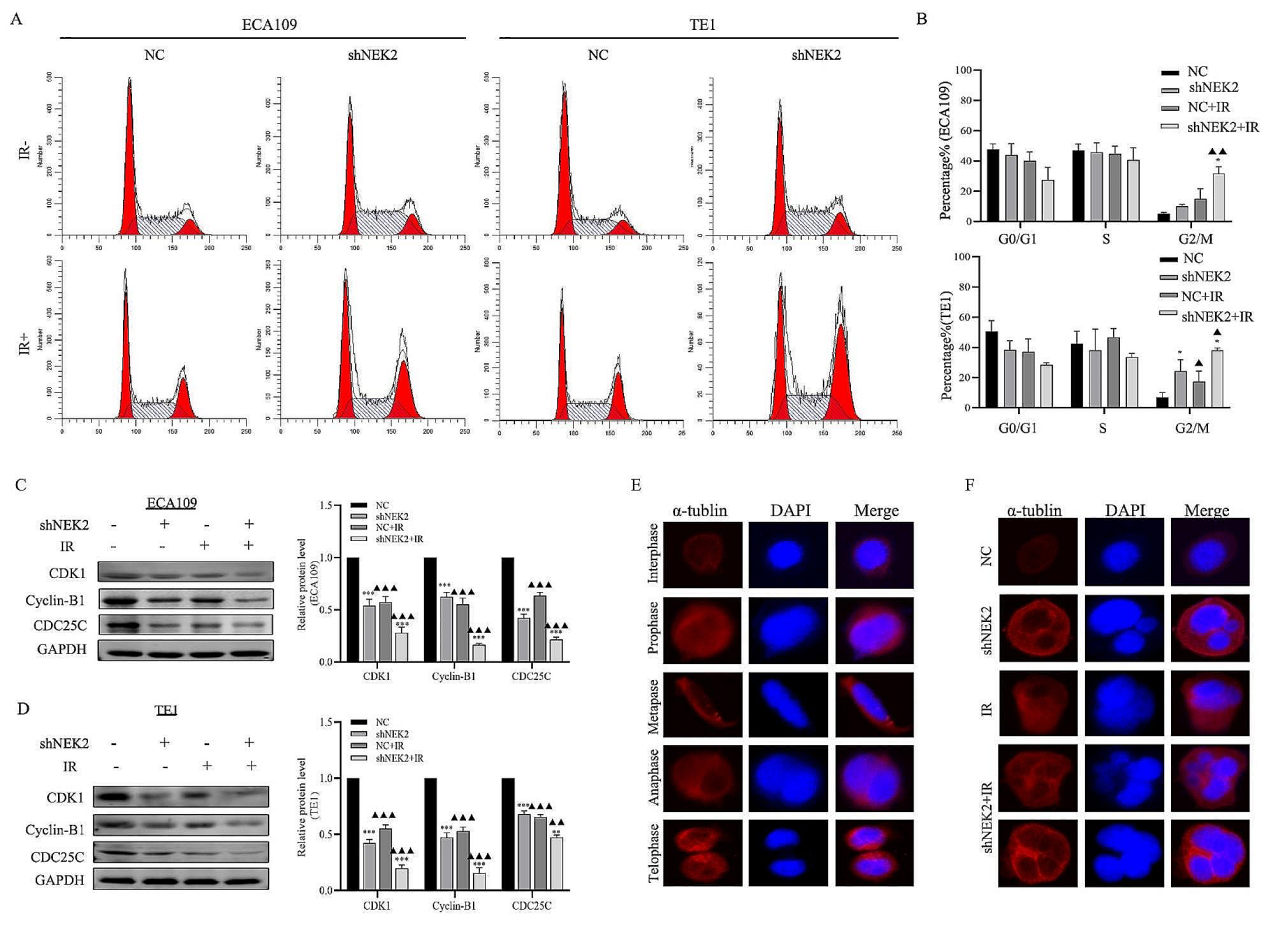
NEK2 knockdown led to cell cycle arrest and spindle microtubules confusion of ECA109 and TE1 ESCC cells. **A, B** Flow cytometry analysis and quantified histogram of cell cycle distribution. **C**, **D** shNEK2 regulated cell cycle proteins to induce G2/M arrest after IR. **E** Changes in morphology and nucleus of mitosis process in ECA109 cells. **F** ECA109 cells were treated with shNEK2 and IR led to spindle microtubules confusion, which contributed to chromosome dissociation abnormality. * *p* < 0.05. A comparison with the corresponding non-irradiated group is indicated by triangles, ^▲^*p* < 0.05, ^▲▲^*p* < 0.01

It was essential to determine whether cell cycle distribution would be altered along with NEK2 knockdown. Flow cytometry analysis showed that NEK2 knockdown resulted in cell cycle arrest in the G2/M phase (Fig. 2A-B). Specifically, treatment with IR significantly increased the percentage of G2/M phase cells in both ECA109 (*p* < 0.05) and TE1 cells (*p* < 0.05) compared to the control group. CDK1, CyclinB1, and CDC25C are important cell cycle related-proteins. The cell cycle-related proteins expression level was investigated by western blot assay. As shown in Fig. 2C-D, the protein levels of CDK1, CyclinB1, and CDC25C in ECA109 and TE1 cells treated with NEK2 knockdown or irradiation were downregulated compared to the control group. Moreover, the group treated with the combination of NEK2 knockdown and irradiation showed a more pronounced G2/M arrest compared to the groups treated with NEK2 knockdown or irradiation alone. These data indicated that NEK2 knockdown can enhance G2/M arrest, and the combined treatment of NEK2 and irradiation maximized G2/M arrest.

In order to investigate the effects of NEK2 on mitosis, immunofluorescence microscopy was used to visualize the mitotic morphology and cell nucleus changes. The abnormal cell division increased significantly after NEK2 knockdown or irradiation treatment (Fig. 2E-F). There were clear abnormalities in chromosome separation during the middle, late, and later stages of mitosis, resulting in an uneven distribution of chromosomes into the two daughter cells. The number of abnormal cell nucleus increased significantly after the combination of NEK2 knockdown and irradiation, including micronucleus, multinucleus and dumbbell nucleus.

#### Increased NEK2 expression enhanced radioresistance of ESCC cells

**Fig. 3:**
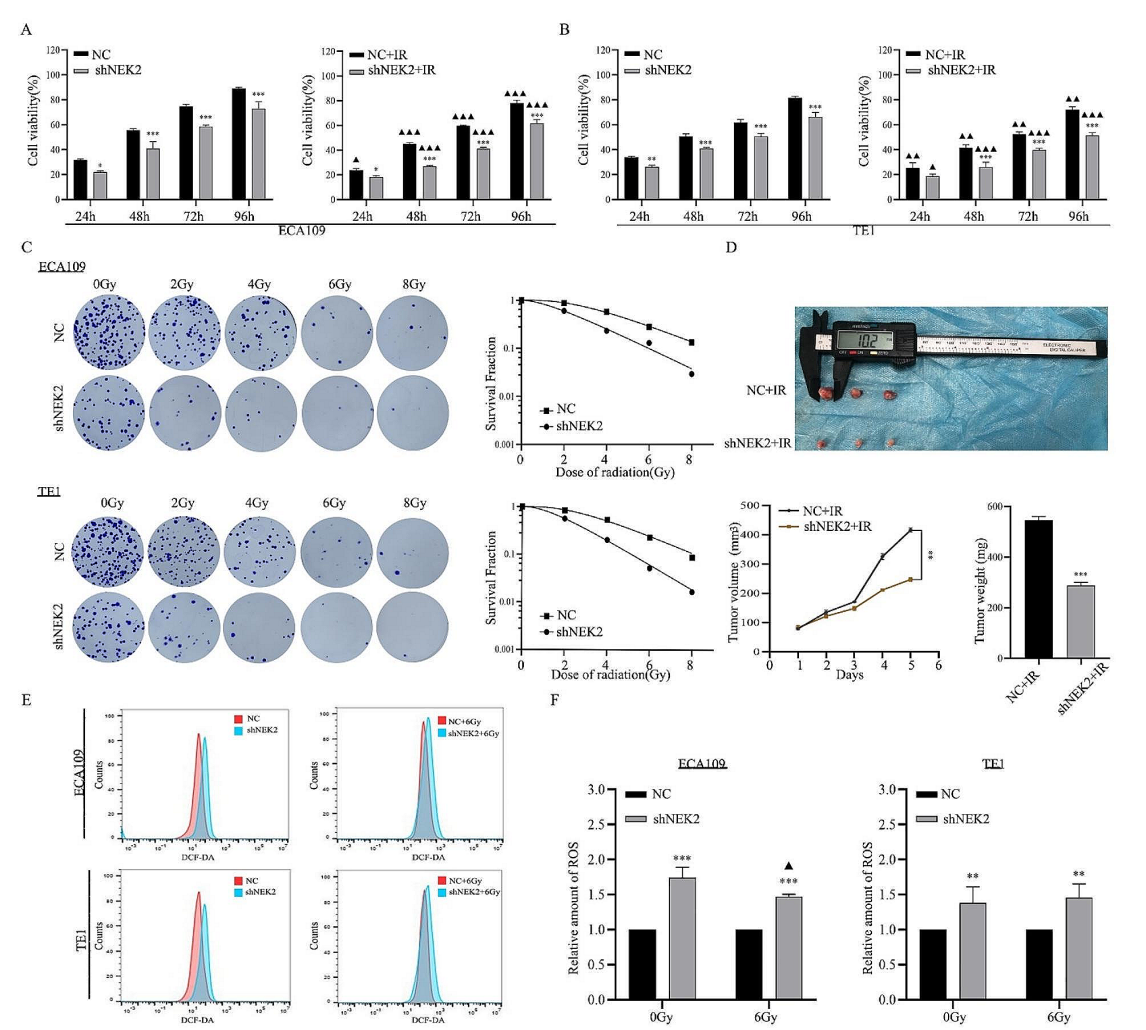
NEK2 knockdown enhanced radiosensitivity of ESCC cells. **A, B** The cell viability was measured by the CCK-8 method after 24, 48, 72 and 96 h with or without 6 Gy X-ray irradiation. **C** Dose responses of survival factions of ECA109 cells and TE1 cells before and after shNEK2 transfection. **D** Tumor xenografts were photographed. Tumor volume and weight were monitored. **E, F** Detection of ROS levels by flow cytometry analysis and histogram in each group. * *p* < 0.05, * *p* < 0.01 and *** *p* < 0.001. A comparison with the corresponding non-irradiated group is indicated by triangles, ^▲^*p* < 0.05, ^▲▲^*p* < 0.01, ^▲▲▲^*p* < 0.001

In order to analyze the correlation between NEK2 and radioresistance in ESCC cells, we constructed the stable knocked down NEK2 expression in ECA109 and TE1 cell lines. CCK-8 assay indicated that the proliferation rates in the NEK2 knockdown group were markedly lower than those in NC group at 48 h, 72 and 96 h with or without irradiation (*p* < 0.05) (Fig. 3A-B). Additionally, colony formation experiments were conducted to analyze the survival differences in colony formation ability upon cells treated with dose-dependent manners. The results showed that the NEK2 knockdown group significantly reduced the number of colonies compared to the NC group after irradiation, suggesting that ESCC cells with NEK2 knockdown exhibited increased radiosensitivity (Fig. 3C). As shown in Fig. 3D, the shNEK2-ECA109 and NC-ECA109 cells were subcutaneously inoculated into nude mice. Compared with the NC-ECA109 cell group, the shNEK2-ECA109 cell group exhibited significantly reduced tumor volume and weight after irradiation (*p* < 0.05).

Given that irradiation can induce the production of cellular ROS in cells, we measured the cellular ROS levels after irradiation and determined whether NEK2 had an effect on regulating cellular ROS accumulation in ESCC cells. As shown in Fig. 3E-F, the NEK2 knockdown increased the ROS level by 1.7-fold in ECA109 cells and 1.4-fold in TE1 cells compared with NC group. Notably, the combination treatment group had more cellular ROS generation at 4 h after 6 Gy irradiation, compared to the IR alone control group. These data revealed that NEK2 knockdown led to ROS generation and alleviated radioresistance.

#### NEK2 knockdown increased IR-induced apoptosis and DNA damage of ESCC cells

**Fig. 4:**
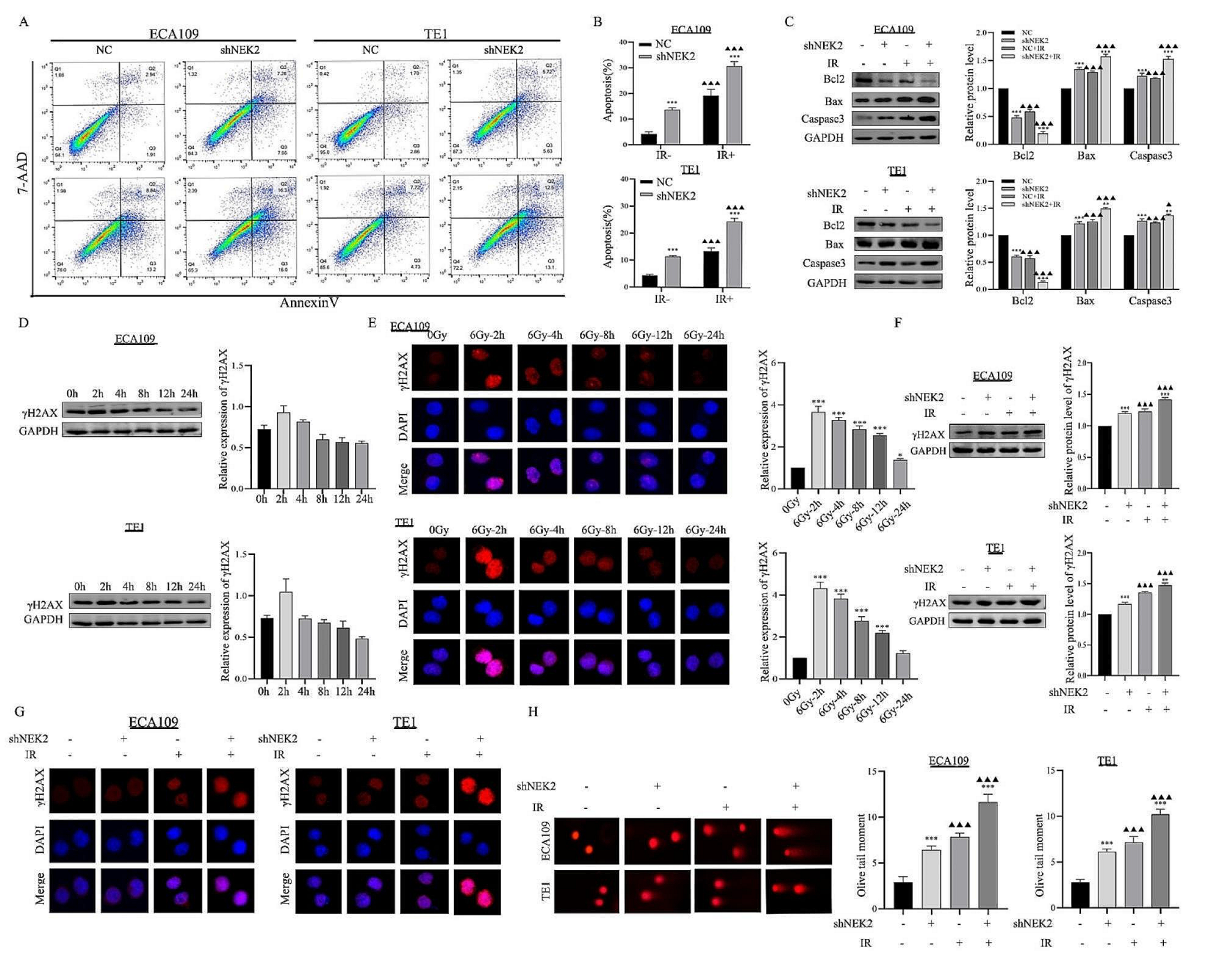
NEK2 knockdown increased IR-induced apoptosis and DNA damage. **A-C** The cell apoptosis rates and apoptosis-related proteins were measured by flow cytometry and western blotting assay. **D** The protein expression of γH2AX varied with time after 6 Gy X-ray irradiation. **E** After irradiation with 6 Gy, ECA109 and TE1 ESCC cells were stained with γH2AX at the different time point (0, 2, 4, 8, 12, 24 h) to observe γH2AX focus formations. **F, G** After different treatment, the protein expression and number of γH2AX focus formations were analyzed. **H** IR plus shNEK2 treatment led to an increased olive tail moment in ESCC cell lines. *** *p* < 0.001. A comparison with the corresponding non-irradiated group is indicated by triangles, ^▲▲▲^*p* < 0.001

The flow cytometry results showed that the proportion of apoptotic cells was 4.26%, 13.65% in NC group and NEK2 knockdown group of ECA109 cells, and 4.36%, 11.47% in NC group and NEK2 knockdown group of TE1 cells (Fig. 4A). Interestingly, compared to single NEK2 knockdown or IR treatment, the combined approach of NEK2 knockdown and IR induced high levels of apoptosis rates in the two ESCC cell lines (Fig. 4B). We also observed an increased trend in Bax and a decreased trend in Bcl2 protein expression following the combined NEK2 knockdown and IR treatment, as well as with NEK2 knockdown or IR treatment alone in both ESCC cell lines (Fig. 4C).

IR-induced DNA double-strand breaks (DSBs) are one of the major contributing factors to the loss of cell viability, and an inability to repair them efficiently can result in cell death [23]. We firstly examined the γH2AX expression, a DNA damage marker, as shown in Fig. 4D. Western blotting showed that γH2AX expression was higher at 2 h after irradiation than that at different time points (0 h, 4h, 8 h, 12 h, and 24 h). It was observed through immunofluorescence that, following irradiation, the expression of the γH2AX protein increased more rapidly compared to un-irradiated cells. These results were consistent with the trend in protein expression (Fig. 4E). The expression of the γH2AX protein remained at a higher level in the combination group compared to ESCC cells receiving only irradiation treatment, suggesting that NEK2 knockdown impeded the DNA repair process, leading to significant unrepaired DNA DSBs (Fig. 4F-G). Additionally, we performed the neutral comet assay on ESCC cells in the shNEK2 and NC groups. The olive tail moment was significantly increased in NEK2 knockdown cells after irradiation (Fig. 4H). These observations indicated that NEK2 knockdown promoted DNA damage, thereby enhancing radiosensitivity.

#### NEK2 induced autophagy in ESCC cells

**Fig. 5:**
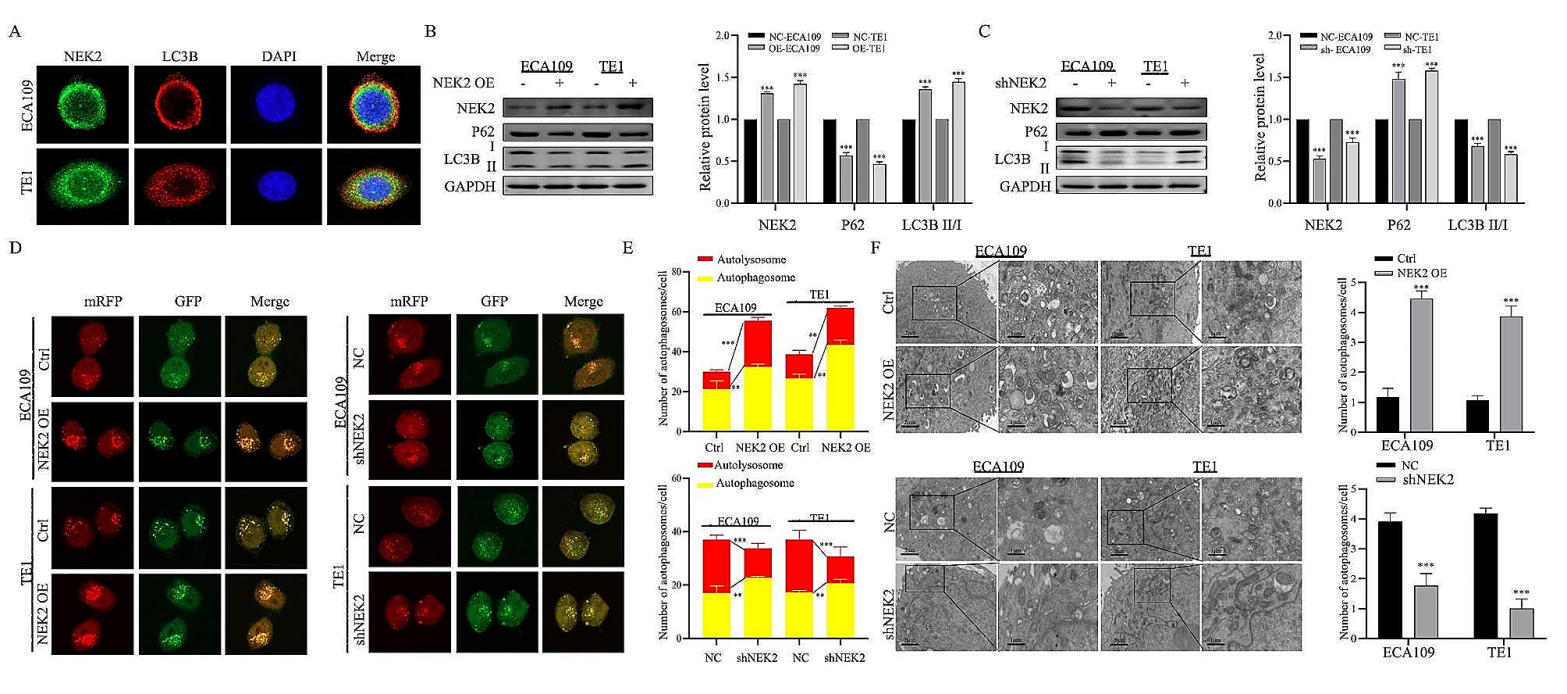
NEK2 modulates autophagy in ESCC cells. **A** Representative immunofluorescence images of NEK2 (Green) and LC3B (Red) protein expression in ECA109 and TE1 ESCC cells. **B, C** Western blotting of NEK2, P62, LC3B and GAPDH in NEK2 OE and shNEK2 ESCC cells. **D** ESCC cells stably transfected with GFP-mRFP-LC3 and treated with NEK2 OE or shNEK2. Laser confocal fluorescence assay (left) and puncta-based quantification (right) for autophagosomes and autolysosomes. In the fluorescence image, red puncta indicate autolysosomes while yellow puncta indicate autophagosomes. Scale bars: 10 μm. **E** The quantified results of autophagosomes and autolysosomes in each group. **F** The autophagosomes of ECA109 and TE1 ESCC cells with NEK2 OE or shNEK2 were determined by transmission electron microscopy (TEM). Scale bar = 2 μm (left) or Scale bar = 1 μm (right). * *p* < 0.05, * *p* < 0.01 and *** *p* < 0.001

To investigate whether NEK2 knockdown increased radiosensitivity by regulating autophagy in ESCC cells, we utilized NEK2 overexpression (OE) cells and shNEK2 cells for analysis. Because the LC3B I and LC3B II are known to represent the indicators for autophagy, we next examined the association between NEK2 expression and LC3B expression in ESCC cells. Immunofluorescence was used to observe the protein expression and localization of NEK2 and LC3B in ESCC cells. As shown in Fig. 5A, NEK2 extensively colocalized with LC3B. We observed an increased the ratio of LC3B II/LC3B I and a decreased level of P62 in NEK2 OE cells compared to control cells. Conversely, NEK2 knockdown led to a significant decrease of LC3B II/LC3B I in the same ESCC cell lines (Fig. 5B-C). As shown in Fig. 5D-E, increased autophagosome formation was observed in NEK2 OE ECA109 and TE1 cells, but it was reduced in ESCC cells from shNEK2 group. Consistent with these changes, TEM data revealed that the double-membrane autophagosomes accumulated in the NEK2 OE ESCC cells and decreased in shNEK2 group ESCC cells (Fig. 5F). These results showed that NEK2 could regulate the process of autophagy in ESCC cells.

### NEK2 knockdown inhibited autophagy in IRtreated ESCC cells

**Fig. 6:**
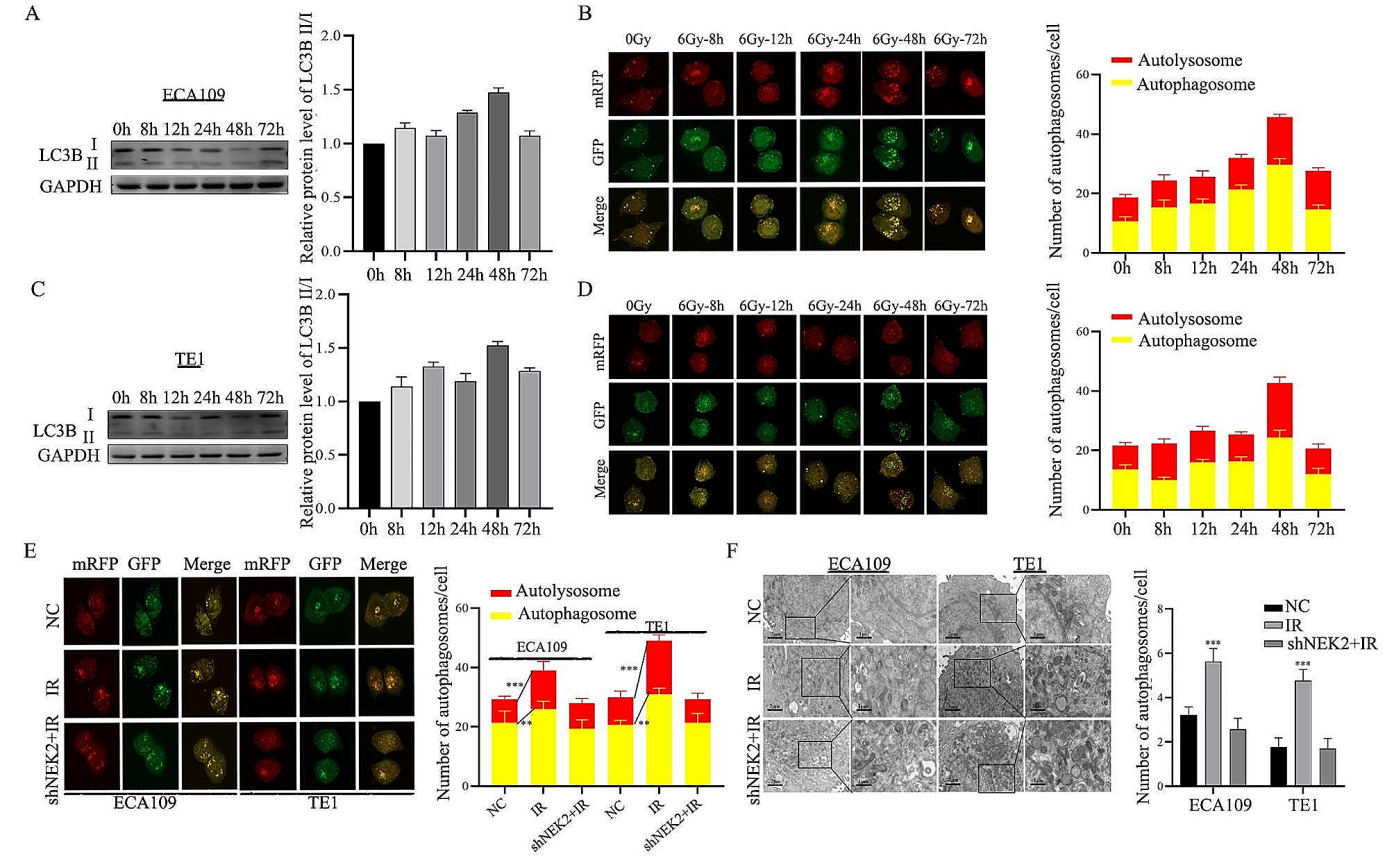
NEK2 knockdown inhibits autophagosome accumulation in IRtreated ESCC cells. **A, B** After irradiation with 6 Gy, autophagosome formation was determined at different time points (0 h, 8 h, 12 h, 24 h,48 h, 72 h) in ECA109 ESCC cells. **C, D** After irradiation with 6 Gy, autophagosome formation was determined at different time points (0 h, 8 h, 12 h, 24 h,48 h, 72 h) in TE1 ESCC cells. **E, F** Immunofluorescence and transmission electron microscopy (TEM) results of ECA109 and TE1 ESCC cells treated with shNEK2, IR or IR plus shNEK2. *** *p* < 0.001

After treatment with irradiation, the expression of the LC3B protein was significantly increased at 48 h in ECA109 cells (Fig. 6A). Simultaneously, a fluorescence assay observed an increase in yellow puncta at 48 h, indicating increased autophagosome formation (Fig. 6B). Consistently, the ratio of LC3B II/LC3B I and autophagosomes formation at 48 h were higher than at different time points in TE1 cells (Fig. 6C-D). Irradiation significantly increased the number of fluorescent autophagosomes and double-membrane autophagosome structures compared to the NC group, but IR plus shNEK2 treatment can attenuate this trend (Fig. 6E-F). These data suggested that NEK2 knockdown inhibited autophagy in IRtreated ESCC cells.

### Activation of autophagy reversed the NEK2 knockdown-induced radiosensitization

**Fig. 7:**
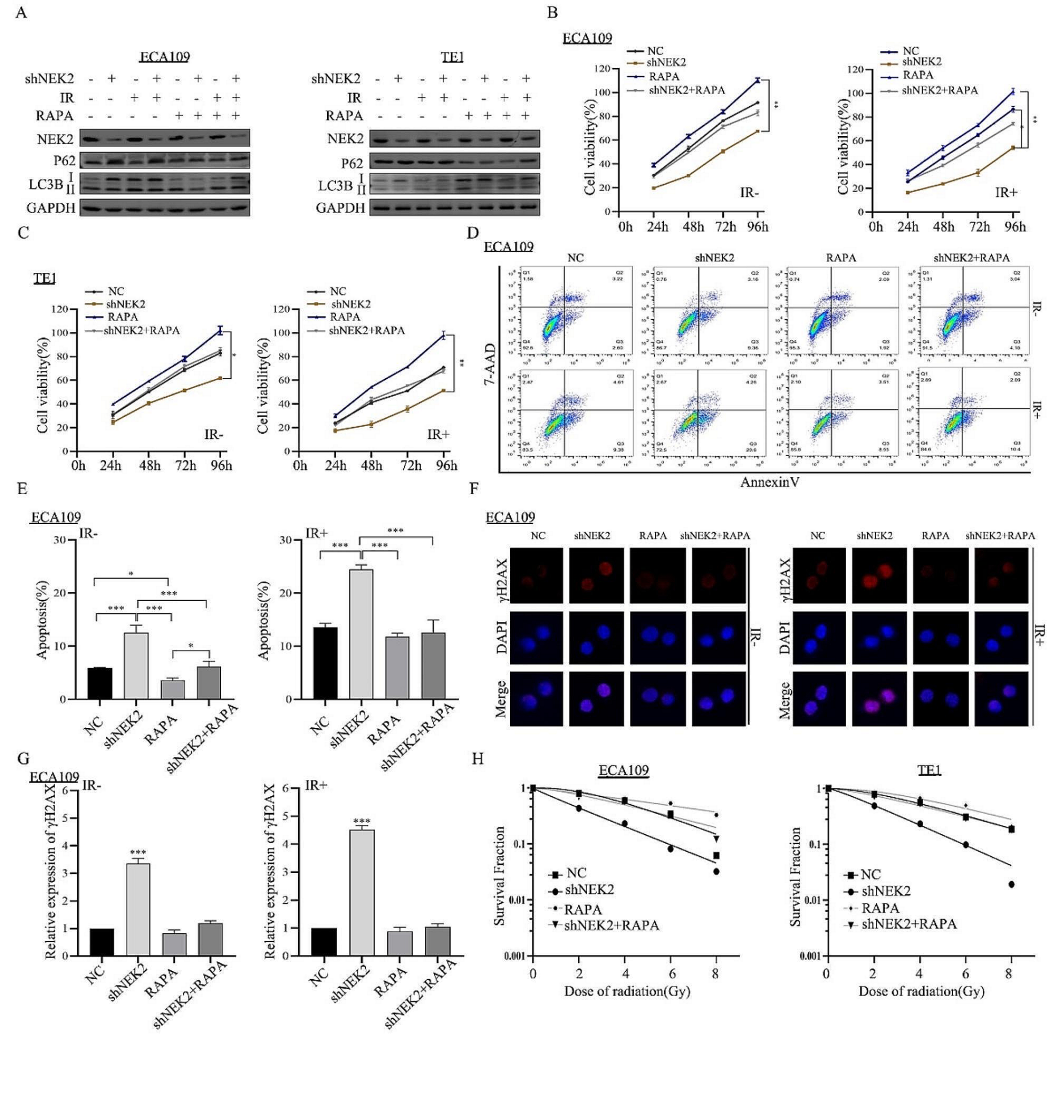
Autophagy activation decreases the NEK2 knockdown-induced radiosensitization. **A** Protein expression levels of P62 and LC3B with or without RAPA were detected by western blotting assay. **B, C** The cell viability was measured by the CCK-8 method after 24, 48, 72 and 96 h in different treatment groups. **D, E** After treatment with RAPA, flow cytometric analysis was conducted to observe cell apoptosis rates. **F, G** Immunofluorescence and quantified histogram was used to analyze the γH2AX focus formations in shNEK2-transfected ECA109 cells treated with RAPA. **H** Dose responses of survival factions were analyzed in different groups. * *p* < 0.05, * *p* < 0.01 and *** *p* < 0.001

Our data has shown that NEK2 was involved in the autophagy of ESCC cells. Subsequently, we aimed to investigate whether autophagy was the crucial mechanism regulating NEK2-mediated radiosensitivity. Rapamycin (RAPA), a specific activator of autophagy, was used to treat ECA109 and TE1 cells. We observed an increased LC3B II/LC3B I ratio and decreased P62 levels in shNEK2 ESCC cells after RAPA treatment (Fig. 7A), and autophagy recovery was also confirmed after irradiation. After 6 Gy X-ray irradiation, CCK-8 assays revealed that treating cells with shNEK2 and RAPA alleviated the decrease in cell viability induced by shNEK2 transfection. Importantly, the cell viability treated with RAPA alone was increased (Fig. 7B-C). Further, the flow cytometry results showed that treating with shNEK2 and RAPA alleviated the apoptosis of ECA109 cells compared to the shNEK2 group (Fig. 7D-E). Immunofluorescence assays and histograms revealed that RAPA treatment reduced IR-induced expression of γH2AX. However, the effect was weakened by NEK2 knockdown (Fig. 7F-G). After treating with various dose irradiation, the colony formation experiment was performed to confirm the recovery effect using the RAPA. The results showed that ESCC cells exhibited a certain level of radioresistance when treated with RAPA compared to treatment with irradiation alone, indicating that the radioresistance was associated with autophagy (Fig. 7H).

## NEK2 induced autophagy through TRIM21 in ESCC cells

**Fig. 8:**
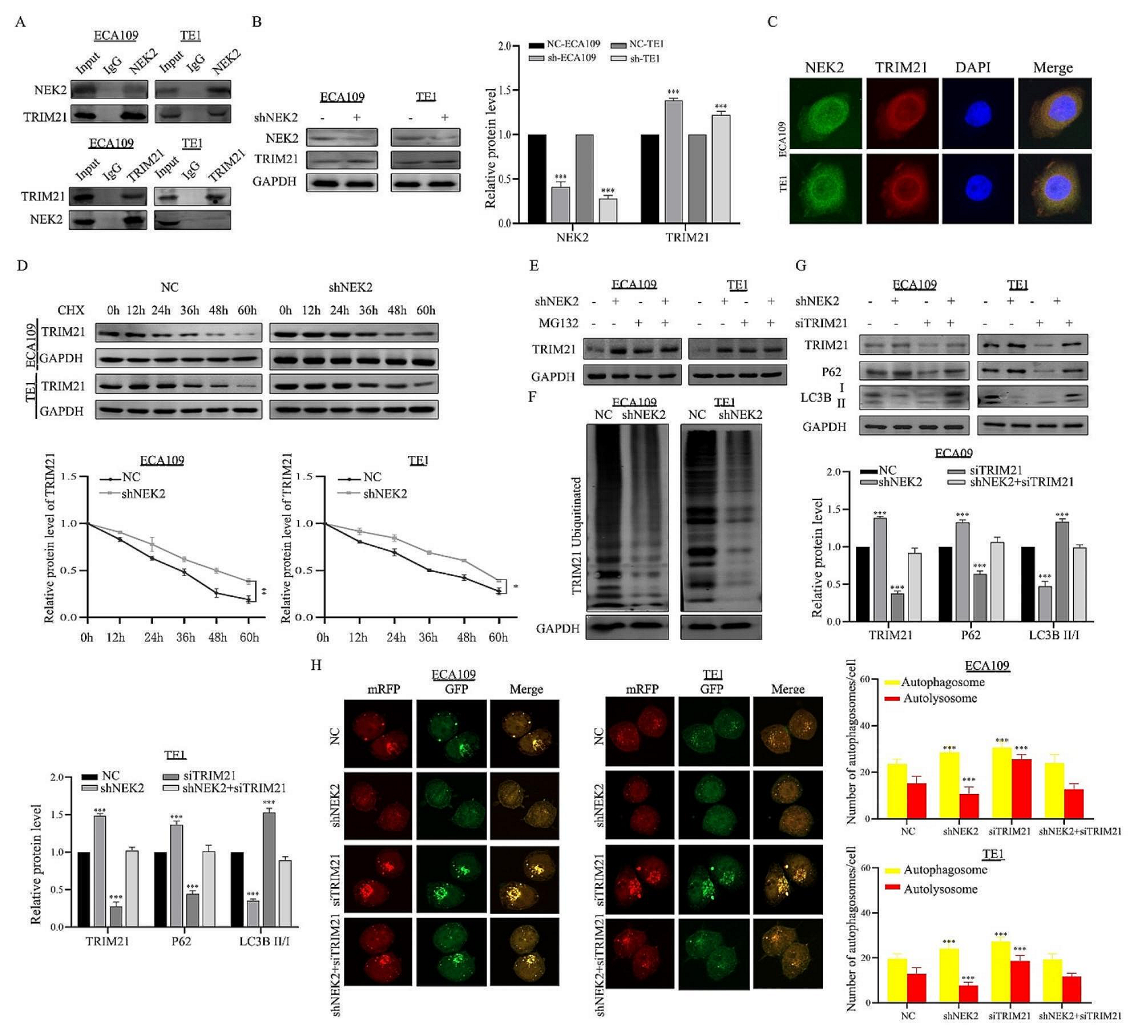
NEK2 knockdown inhibits autophagy through elevating TRIM21 protein expression. **A** The interaction between NEK2 and TRIM21 was proved by means of coimmunoprecipitation. NEK2 antibody and TRIM21 antibody were used to pull down interacting proteins, respectively. **B** The regulatory relationship was confirmed by western blotting. **C** Colocalization analysis of NEK2 (green) and TRIM21 (red) by laser confocal fluorescence microscopy. **D** ECA109 and TE1 ESCC cells were treated with cycloheximide (CHX, 10 µg/ml), and the protein expression levels of TRIM21 and GAPDH were analyzed by western blotting. **E** ECA109 and TE1 ESCC cells were treated with MG132 (10 µM) for 6 h, and the western blotting was conducted using anti-TRIM21 and anti-GAPDH antibodies. **F** The effect of NEK2 knockdown on the ubiquitin level of TRIM21 by Co-IP and western blotting. **G** After transfection with siTRIM21, the expression of TRIM21, P62 and LC3B in ECA109 and TE1 ESCC cells by western blotting. **H** After transfection with siTRIM21, accumulation of autophagosomes and autolysosomes was observed by laser confocal fluorescence assay, Scale bar: 10 μm

To identify the molecules involved in NEK2-mediated autophagy, Co-IP was performed to identify the NEK2-associated proteins in ESCC cells. When compared with proteins pulled down by normal mouse IgG, the silver-stained gel revealed a significant band difference when using NEK2 antibodies. Consequently, we conducted Western blotting analysis and identified TRIM21 as a candidate protein. The results confirmed that both NEK2 and TRIM21 were detectable in proteins immunoprecipitated with NEK2 antibodies and TRIM21 antibodies, but not in the corresponding IgG control (Fig. 8A). We further investigated whether TRIM21 expression level was regulated by NEK2. Western blotting results revealed a negative association between NEK2 and TRIM21, with TRIM21 expression upregulating upon NEK2 knockdown. (Fig. 8B). Additionally, immunofluorescence results found that NEK2^’^s subcellular localization was consistent with the TRIM21 protein in ECA109 and TE1 cells (Fig. 8C). These results confirmed that NEK2 stably interacted with TRIM21 and can regulate TRIM21 expression. Interestingly, adding the protein synthesis inhibitor (cycloheximide) to NEK2 knockdown ESCC cells significantly extended the half-life compared to the NC group (Fig. 8D). The expression level of TRIM21 in the NEK2 knockdown group was significantly higher than in the NC group. However, this trend was attenuated by treatment with the proteasome inhibitor MG132 (10 µM) (Fig. 8E). We also assessed the ubiquitination level of TRIM21 in NEK2 knockdown ECA109 and TE1 cell lines using Co-IP with the TRIM21 antibody. Consistent with these results, NEK2 knockdown inhibited the ubiquitination level of TRIM21 (Fig. 8F). All together, these findings supported our hypothesis that NEK2 plays an essential role in the regulation of TRIM21.

Since NEK2 regulated the stability of TRIM21, we hypothesized that the TRIM21 was involved in NEK2-mediated autophagy. To test this hypothesis, siRNAs targeting TRIM21 were transfected into ECA109 and TE1 ESCC cells. Western blotting assay was conducted to detect the protein expression levels of TRIM21, P62, LC3B I, and LC3B II (Fig. 8G). The results showed that targeting TRIM21 siRNA notably induced the expression of LC3B II and decreased P62 level. Importantly, NEK2 and TRIM21 double knockdown presented higher LC3B II expression compared to NEK2 knockdown alone, indicating that autophagy inhibition induced by NEK2 knockdown was attenuated by TRIM21 knockdown. Similarly, immunofluorescence data was consistent with western blotting assay results. The results also demonstrated that knockdown of TRIM21 significantly increased autophagosome formation induced by NEK2 (Fig. 8H). The above results demonstrated that NEK2 enhanced autophagy through regulating TRIM21.

### Knockdown of TRIM21 reversed NEK2-mediated radioresistance in ESCC cells

**Fig. 9:**
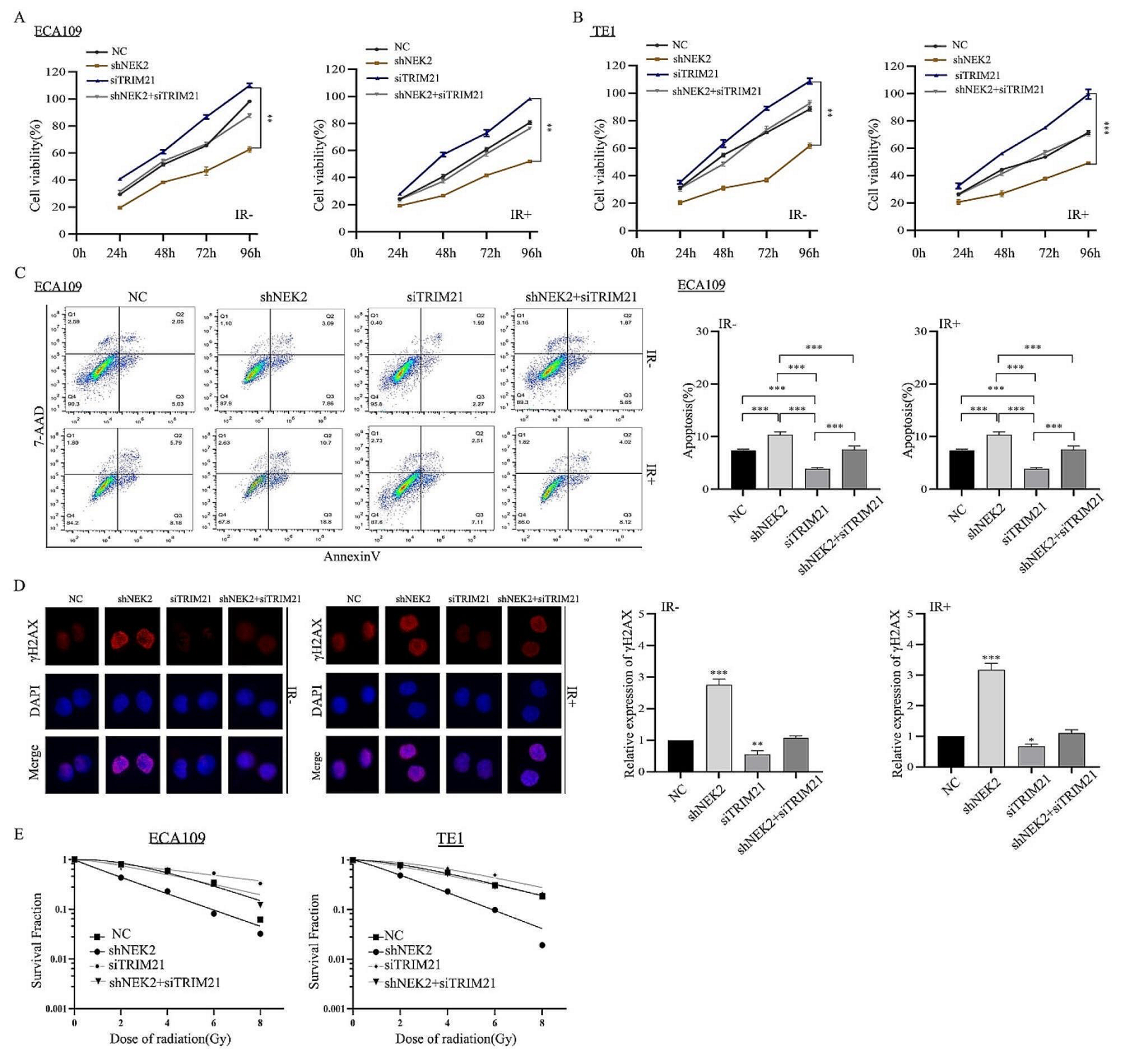
NEK2 increases radioresistance of ESCC cells through TRIM21. **A, B** After transfection with siTRIM21, the ECA109 and TE1 ESCC cells viability was measured by the CCK-8 method after 24, 48, 72 and 96 h. **C** After transfection with siTRIM21, flow cytometric assay was performed to measure the cell apoptosis rates. **D** Immunofluorescence assays showing the effect of TRIM21 on the expression of γH2AX focus formations in ECA109 cells treated with siTRIM21. **E** Quantitation of clonogenic assay to examine the effects of different X-ray IR doses on cell viability of ECA109 and TE1 ESCC cells with TRIM21 knockdown. * *p* < 0.05, * *p* < 0.01 and *** *p* < 0.001

The data described above indicated that NEK2 knockdown enhanced autophagy-mediated radiosensitization of ESCC cells, potentially inducing autophagy through TRIM21. Based on our current data, we further investigated whether NEK2 knockdown promoted radiosensitization by regulating TRIM21 expression. TRIM21 was knocked down in shNEK2 ESCC cells and control cells, followed by X-ray irradiation treatment. CCK8 results indicated that the ability of NEK2 knockdown ESCC cells was weaker than that of control cells after irradiation treatment, but knockdown of TRIM21 in shNEK2 cells notably increased viability after irradiation (Fig. 9A-B). Interestingly, we examined cell apoptosis rates in NEK2 and TRIM21 double knockdown ESCC cells. The results showed that increased apoptotic rates were observed in shNEK2 cells compared to cells expressing NEK2 and TRIM21 double knockdown after X-ray irradiation treatment, indicating that TRIM21 knockdown reversed the radiosensitization in shNEK2 cells (Fig. 9C). Subsequently, immunofluorescence results showed that the NEK2 and TRIM21 double knockdown resulted in decreased expression of γH2AX comparison to shNEK2 cells (Fig. 9D). After knockdown of TRIM21, the colonies capacity of shNEK2 ECA109 and TE1 cells significantly increased when compared to the control groups following treatment with the same dose of X-ray irradiation (Fig. 9E).

## Discussion

**Fig. 10:**
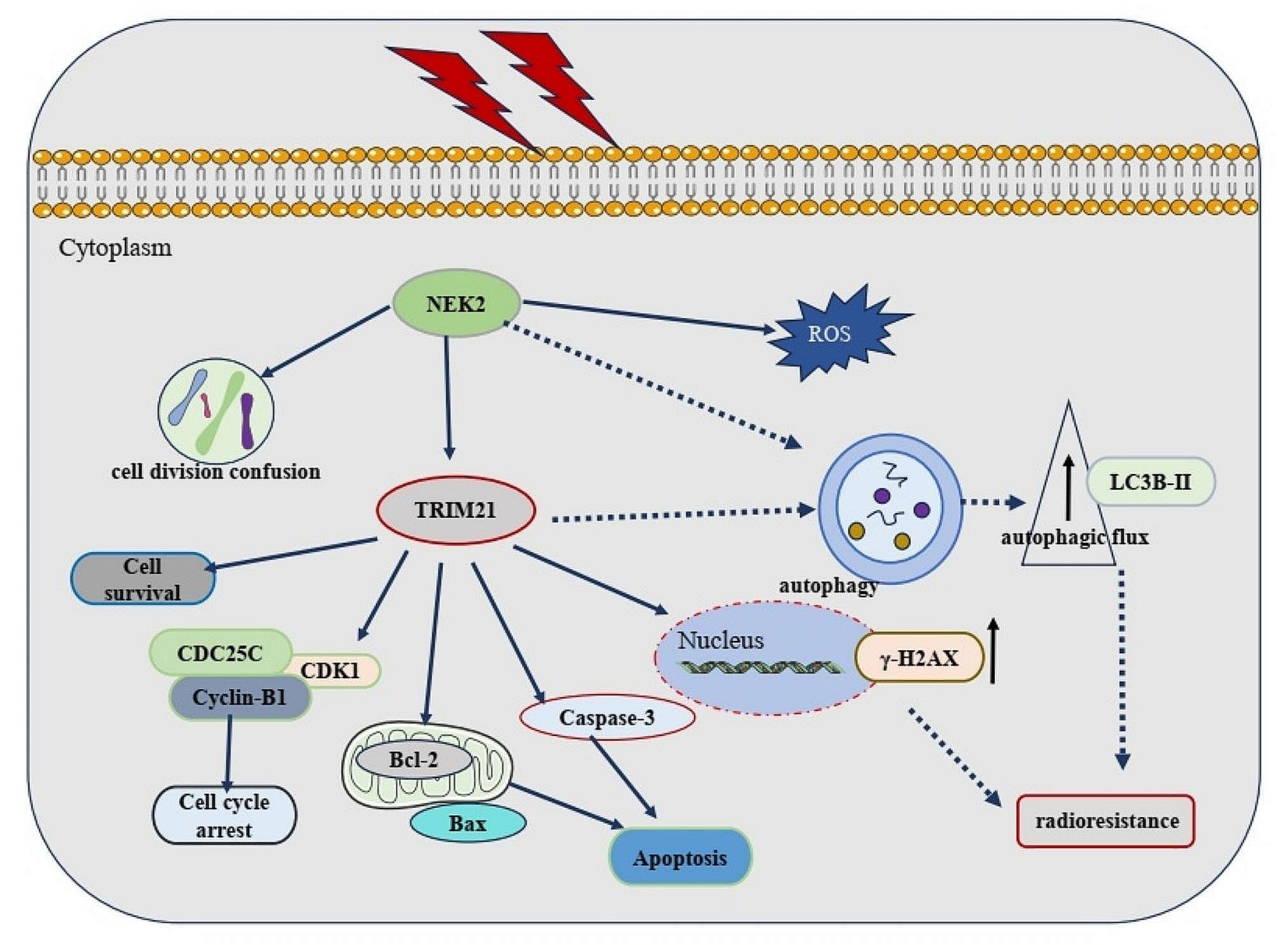
A mechanism model of NEK2 regulating ESCC radioresistance by TRIM21-medicated autophagy. NEK2 is overexpressed in ESCC cells. It regulates the TRIM21 expression and facilitates the protective autophagy, thus promoting ESCC malignant progression. Meanwhile, NEK2 induced cell cycle arrest, apoptosis, ROS production and DNA damage, which, in turn, further promotes radioresistance in ESCC cell lines

Radiotherapy remains a main modality for ESCC, but radioresistance often leads to local recurrence and makes ESCC refractory. Increasing evidence have confirmed that radioresistance led to poor prognosis after radiotherapy for ESCC patients [24, 25]. The onset of radioresistance is poorly understood, and the underlying molecular mechanism are not fully elucidated. Hence, understanding how cancers acquire intrinsic or acquired resistance to X-ray irradiation is a major issue that needs to be addressed. In this study, we identified the antitumor effects of NEK2 in mediating cell cycle, apoptosis activity, ROS levels, DNA damage, and tumor progression, contributing to radioresistance in ESCC. Intriguingly, we found NEK2 could induce autophagy through TRIM21, which contributed to radioresistance in ESCC cells. These findings confirmed that targeting NEK2 may be a promising approach for enhancing anticancer efficacy of radiotherapy against ESCC (Fig. 10).

NEK2, a cell-cycle–regulated protein kinase localized in the centrosome, has been closely correlated with an aggressive phenotype and prognosis in various cancers [26, 27]. Recently, Lee et al. demonstrated that targeting NEK2 with siRNA worked synergistically with paclitaxel and doxorubicin by promoting apoptosis in triple-negative breast cancer cells, thereby enhancing anticancer chemotherapy sensitivity [28]. Xu et al. indicated that downregulation of NEK2 in human nasopharyngeal carcinoma cell lines significantly inhibited invasive cellular phenotypes and increased the sensitivity of cells to cisplatin [29]. Of note, Xu et al. initially confirmed that targeting NEK2 improves the response to radiotherapy in cervical cancer through in vitro and in vivo models. This result suggested that this anti-tumor strategy could potentially address the constraints of radiotherapy and offer promise for the clinical management of cervical cancer patients [15]. These findings confirmed a promising role of NEK2 in beneficial radiobiological effects. However, the relationship between NEK2 and ESCC cell radiotherapy response, as well as the involved molecular mechanisms, are currently unknown. In the present study, we have, for the first time, proved that NEK2 was associated with radiotherapy response of ESCC cells after X-ray irradiation. The results confirmed that NEK2 knockdown can induce the G2/M arrest, high levels of apoptosis rates, ROS production, and DNA damage, all of which reversed radiotherapy resistance in ESCC cells. Thus, NEK2 is expected to be a potential therapeutic target in ESCC patients exhibiting high NEK2 expression in tumor cells.

Radioresistance is involved with multiple biological mechanisms. Radiotherapy can cause damage to the structure of extranuclear cells, and then damage can lead to the accumulation of metabolites, changes in mitochondrial membrane potential, ROS production, and the activation of stress signaling pathways to facilitate autophagy. Autophagy is a multi-step biological catabolic mechanism in which cells can remove damaged proteins and organelles to facilitate cellular homeostasis [30, 31]. Physical disorder or autophagy-related proteins knockdown resulted in autophagy inhibition, which may facilitate abnormal aggregation of ubiquitinated protein to decrease the vitality of cells. Thus, autophagy-mediated protein degradation system is essential for ESCC cells to maintain homeostasis. Autophagy present sophisticated roles during tumor treatment. Previous study confirmed that radiotherapy can induce cancer cell autophagy, which enhanced radioresistance [32]. Silencing auto-lysosomal genes in lung cancer cells resulted in a significant radiosensitisation [33]. Radioresistant breast tumor cells showed a strong reduction of autophagy and enhanced the radiosensitivity after treatment with autophagy inhibitors 3-MA or chloroquine (CQ) [34]. In addition, inhibition of autophagy mediated by HMGB1 [35], LMP1 [36], or NEDD8 [37] also promoted treatment responses to IR in ESCC, nasopharyngeal carcinoma and oral squamous cell carcinoma, respectively. Moreover, Furthermore, the induced formation of autophagy in esophageal cancer cells could lead to radioresistance [38]. Importantly, our data showed that inhibition of autophagy by NEK2 knockdown can increase radiosensitivity in ESCC cells; in contrast, induced formation of autophagy by NEK2 OE triggered autophagy activity, leading to radioresistance. These results clearly confirmed the role of autophagy in mediating response to radiotherapy, indicating that inhibition of IR-induced protective autophagy would improve anti-tumor benefits.

TRIM protein family member Tripartite motif-containing protein 21 (TRIM21) is universally expressed in various cells and is involved in innate immune responses [39]. The recent study has shown that TRIM21 has been recognized to be involved in the regulation of autophagy [40, 41]. More specifically, the role of TRIM21 in tumor autophagy is remains controversial. It can either exhibit pro-autophagic or inhibit autophagy by targeting and degrading various proteins in the autophagy-related pathway. For example, Zhang et al. showed TRIM21 can promote osteosarcoma cell autophagy via enhancing the autophagic flux and the degradation of P62 [42]. Kimura et al. confirmed that TRIM21 can facilitate autophagy by regulating the core proteins (ULK1, BECN1, and P62) of autophagy process in the cells and acts as an autophagic receptor [43]. Conversely, there are still some reports indicating that TRIM21 can inhibit the autophagy process in tumor cells. Chen et al. reported that TRIM21 overexpression resulted in decreased autophagic flux and increased bort-induced multiple myeloma cell apoptosis by ubiquitinating ATG5 [44]. In view of these data, we investigated whether high TRIM21 protein expression in ESCC cells inhibited autophagy to enhance radiosensitivity. Thus, we performed western blotting and immunofluorescence experiments to assess a range of crucial autophagy genes and changes in autophagic flux. In the present study, our data showed that NEK2 bound to TRIM21, and NEK2 knockdown led to an upregulation of TRIM21 protein levels. Western blotting results showed that TRIM21 knockdown increased the expression of LC3B II expression but decreased P62 expression. Immunofluorescence results of TRIM21 knockdown in ESCC cells showed a strong autophagic flux, suggesting the negative regulatory functions of TRIM21 in autophagy.

## Conclusions

In summary, our findings provided deep insights into the radioresistance of NEK2 in ESCC, elucidating the significant clinical implications of NEK2 knockdown as a promising therapeutic strategy for ESCC. NEK2 negatively regulated the activation of the autophagy, and TRIM21-mediated autophagy regulation was dependent on NEK2. As a result, NEK2 acted by targeting TRIM21 to enhance autophagy and promoted radioresistance in ESCC cells. Therefore, combining X-ray irradiation with NEK2 targeting and autophagy inhibitors, this triple-combination therapy may represent a viable option to overcome radioresistance in ESCC treatment.

## Data Availability

No datasets were generated or analysed during the current study.

## Abbreviations

RT: Radiotherapy
ESCC: esophageal squamous cell carcinoma
NEK2: NIMA-related kinase 2
HEEC: Human esophageal epithelial cells
CCK8: Cell Counting Kit-8
TEM: Transmission electron microscopy
TRIM21: Tripartite motif-containing protein 21
LC3B II: Light chain 3 beta 2
EMT: Epithelial-mesenchymal transition
IR: Ionizing radiation
GEO: Gene Expression Omnibus
OS: Overall survival
FBS: Fetal bovine serum
PVDF: Polyvinylidene fluoride
MCE: Med Chem Express
ROS: Reactive Oxygen Species
DSBs: DNA double-strand breaks
SD: Standard deviation
RAPA: Rapamycin
qRT-PCR: Quantitative real-time PCR
Co-IP: Co-immunoprecipitation
CQ: Chloroquine
